# Not Just a Bystander: The Emerging Role of Astrocytes and Research Tools in Studying Cognitive Dysfunctions in Schizophrenia

**DOI:** 10.3390/ijms22105343

**Published:** 2021-05-19

**Authors:** Chia-Yuan Chang, Da-Zhong Luo, Ju-Chun Pei, Ming-Che Kuo, Yi-Chen Hsieh, Wen-Sung Lai

**Affiliations:** 1 Department of Psychology, National Taiwan University, Taipei 10617, Taiwan; d03227103@ntu.edu.tw (C.-Y.C.); d06227102@ntu.edu.tw (D.-Z.L.); lagipei@gmail.com (J.-C.P.); r08227134@ntu.edu.tw (Y.-C.H.); 2 Neurobiology and Cognitive Science Center, National Taiwan University, Taipei 10617, Taiwan; kuomingche0402@gmail.com; 3 Department of Neurology, National Taiwan University Hospital, Taipei 100225, Taiwan; 4 Graduate Institute of Brain and Mind Sciences, National Taiwan University, Taipei 10617, Taiwan

**Keywords:** astrocytes, schizophrenia, cognitive dysfunction, glutamate transmission, glutamate trisynapse, blood–brain barrier, animal models, behavioral tasks

## Abstract

Cognitive dysfunction is one of the core symptoms in schizophrenia, and it is predictive of functional outcomes and therefore useful for treatment targets. Rather than improving cognitive deficits, currently available antipsychotics mainly focus on positive symptoms, targeting dopaminergic/serotoninergic neurons and receptors in the brain. Apart from investigating the neural mechanisms underlying schizophrenia, emerging evidence indicates the importance of glial cells in brain structure development and their involvement in cognitive functions. Although the etiopathology of astrocytes in schizophrenia remains unclear, accumulated evidence reveals that alterations in gene expression and astrocyte products have been reported in schizophrenic patients. To further investigate the role of astrocytes in schizophrenia, we highlighted recent progress in the investigation of the effect of astrocytes on abnormalities in glutamate transmission and impairments in the blood–brain barrier. Recent advances in animal models and behavioral methods were introduced to examine schizophrenia-related cognitive deficits and negative symptoms. We also highlighted several experimental tools that further elucidate the role of astrocytes. Instead of focusing on schizophrenia as a neuron-specific disorder, an additional astrocytic perspective provides novel and promising insight into its causal mechanisms and treatment. The involvement of astrocytes in the pathogenesis of schizophrenia and other brain disorders is worth further investigation.

## 1. Schizophrenia and Unmet Needs in the Treatment of Schizophrenia

Schizophrenia is a severe mental disorder that affects approximately 1% of the world’s population and places a great financial burden on our health systems, families, and societies around the world [1]. This debilitating brain disorder typically emerges in late adolescence and early adulthood. Accumulating evidence suggests that genes, environmental insults, and brain changes contribute to the etiopathogenesis of this psychiatric disorder. Major symptoms of schizophrenia include positive symptoms (e.g., hallucinations, delusions, disorganized speech and behaviors), negative symptoms (e.g., blunted affect, alogia, anhedonia, asociality, avolition, poverty of speech), and cognitive deficits (e.g., thought disorders, working memory dysfunction, executive dysfunction, attentional deficits) [2,3]. Schizophrenia is a long-lasting brain disorder that distorts the way a person thinks, behaves, perceives reality, expresses emotions, and relates to others.

Similar to many other neuropsychiatric disorders, the etiology and pathophysiology of schizophrenia remain unclear. Antipsychotic medications (also known as neuroleptics) are the mainstay of pharmacological treatment for patients with schizophrenia and other psychotic disorders, including schizoaffective disorder, delusional disorder, and bipolar affective disorder, and the major targets of these drugs are positive symptoms of schizophrenia [4]. In contrast, negative symptoms of schizophrenia can often linger or worsen over time, accompanied by impaired cognitive function, such as working memory, executive function, and decision making. Current market antipsychotics have mainly focused on positive and mood-related symptoms, targeting dopaminergic and serotoninergic neurons [5]. In addition, a substantial portion of patients also suffer treatment-resistant positive symptoms, which cannot be improved by even second-generation antipsychotic agents (e.g., clozapine). Cognitive dysfunction is a core feature of schizophrenia that predicts functional outcome and treatment adherence [6]. However, cognitive deficits in schizophrenia have not gained much attention until recently, and available antipsychotic medications are relatively ineffective in improving negative and cognitive deficits. In contrast to the conventional view of dopamine involvement in schizophrenia (i.e., dopamine hypothesis of schizophrenia), other neurotransmitter systems (e.g., glutamatergic and GABAergic neurotransmissions) and glial cells have gradually gained increasing attention in the investigation of the pathophysiology and treatment of schizophrenia in recent decades. The hypothesis that higher cognitive function solely comprises the integrated product of neuronal activity has been challenged by evidence showing the importance of glial cells to both the development and structure of local neural networks.

## 2. The Emerging Role of Astrocytes in Schizophrenia

Traditionally, the importance and significance of glia have long been unappreciated and ignored, even though up to 90% of the cells in our brain are glial cells, not neurons. Astrocytes, whose name is derived from Greek and means “star-like cells” are a subtype of glial cells that are active dynamic signaling players in the central nervous system (CNS). Astrocytes participate in a variety of essential physiological processes in the brain, such as the formation and maturation of synapses, receptor trafficking, control of the homeostasis of ions and energy metabolites, clearance of neurotransmitters, formation of the blood–brain barrier (BBB), provision of nutrients to nervous tissue, and regulation of neurogenesis and brain wiring [7]. They also play an important role in the regulation of synaptic activity, plasticity, neural networks, and cognitive functions. The idea that astrocytes play an essential role in higher cognitive functions and contribute to neuropsychiatric disorders has not gained much attention until recent decades.

### 2.1. Astrocytic Gene and Astrocyte-Related Gene Expression in Schizophrenia

Schizophrenia is a highly polygenic brain disorder. Significant progress has been made over the last decades with genetic association studies and genome-wide association studies (GWAS). The glial hypothesis of schizophrenia assumes that initial disturbances in glial cells (especially astrocytes) can lead to abnormalities in neurons and neurotransmitters, which are involved in the pathogenesis of schizophrenia. Genetic studies have revealed a list of astrocyte-related genes associated with schizophrenia, such as *D-amino acid oxidase* (*DAO*) [8], *excitatory amino acid transporter 2* (*EAAT2*) [9], *excitatory amino acid transporter 4* (*EAAT4*) [10], *S100 calcium-binding protein B* (*S100β)* [11,12,13,14,15], *thrombospondin 1* (*THBS1*) [16], and *serine racemase* (*SR*) [17]. These studies indicate that these individual astrocyte-related genes are potentially associated with schizophrenia. However, it remains unclear whether astrocyte pathology that results from astrocyte-specific de novo genetic mutations or neuronal injury triggers astrocyte dysfunction in patients with schizophrenia. Genetic correlations in human studies alone are not enough to establish causation. Complementary to human studies, animal models play an indispensable and important role in advancing the understanding of the etiopathology of schizophrenia and in the development of new treatments.

In addition, emerging evidence has begun to reveal abnormalities in astrocyte-related gene expression and gene products in patients with schizophrenia. Altered expression of *glial fibrillary acidic protein* (*GFAP*) mRNA and hypertrophic astrocyte morphology were found in postmortem tissue of schizophrenic patients [18,19]. Immunohistochemistry data further confirmed a reduction in GFAP protein levels in the white matter of the cingulate cortex in schizophrenic patients [20]. Increased expression of S100B protein, a calcium binding protein and biomarker for astrocytes and oligodendrocytes, has been found in the cortical brain regions (especially dorsolateral prefrontal cortex) of patients with paranoid schizophrenia compared with controls and patients with residual schizophrenia [21]. A meta-regression analysis further indicated that the expression of S100B is negatively correlated with the total score of the Positive and Negative Syndrome Scale in patients [22]. Furthermore, the mRNA level of *QKI6B*, an isoform of *quaking* (i.e., a gene encoding an RNA-binding protein that is exclusively expressed in glial cells), was upregulated in the prefrontal cortex of patients with schizophrenia [23]. In addition, recent RNA sequencing studies from postmortem brain tissues of schizophrenic patients indicated increased expression of astrocyte-related genes that is independent of medication dosage throughout their lifetime [24]. Thus, findings from these studies support the involvement of astrocytes in the pathogenesis of schizophrenia.

### 2.2. The Role of Astrocytes in Cognitive Deficits in Schizophrenia

In addition to genetic findings of astrocytes in schizophrenia, mounting evidence indicates that astrocytes play a vital role in the maintenance of CNS homeostasis, formation of the BBB, synapse formation, and synaptic glutamate metabolism, which directly or indirectly contribute to the pathogenesis of schizophrenia [15,25,26]. Converging lines of evidence also revealed the impact of astrocyte dysfunction on cognitive deficits in schizophrenia. First, human cortical astrocytes are larger and structurally more complex and diverse than infraprimate mammals and rodents [27,28], suggesting that astrocytic complexity supports increased functional competence of the adult human brain and related cognitive capability. Second, a transgenic mouse with specifically inducible tetanus toxin expression in astrocytes demonstrated that astrocytes are necessary for novel object recognition behavior and the maintenance of functional gamma oscillations in vitro and in awake-behaving mice [29]. Findings from this study also revealed an unexpected role for astrocytes as essential contributors to neural information processing and cognitive function in the brain. Third, to assess the cell-autonomous and species-selective properties of human glia, human glial progenitor cells were engrafted into neonatal immunodeficient mice. Human glial chimeric mice displayed enhanced long-term potentiation and learning performance in Barnes maze navigation, object-location memory, and both contextual and tone fear conditioning [30]. Findings from this study indicated that forebrain engraftment of human glial progenitor cells enhances synaptic plasticity and learning in mice, implying that the role of glial cells in neural processing may have expanded with evolution. Fourth, taking advantage of chemogenetic tools, a recent study demonstrated that astrocytes contribute to remote memory formation by modulating hippocampal–cortical communication during learning [31]. Last but importantly, human iPSC glial mouse chimeras were created and revealed a causal role for impaired glial maturation in the development of schizophrenia [32]. In this study, humanized glial chimeric mice were produced from iPSCs derived from patients with childhood-onset schizophrenia to investigate whether intrinsic glial dysfunction contributes to the pathogenesis of schizophrenia. The schizophrenia glial mice showed delayed astrocytic differentiation and abnormal astrocytic morphologies as well as reduced prepulse inhibition and abnormal behaviors. Thus, these studies not only indicate the essential role of astrocytes in neural information processing and cognitive functions but also support the involvement of glial dysfunction in the pathogenesis of schizophrenia. The consequence of astrocyte dysfunction could result in abnormal neurotransmitter release (especially glutamatergic neurotransmission) and cognitive function impairment in schizophrenia. The precise underlying mechanism is worth further investigation. Here, we simply highlight two fundamental functions of astrocytes (i.e., BBB formation and modulation of synapse transmission) and their potential roles in the etiopathology of schizophrenia.

### 2.3. Astrocytic Modulation of the BBB in Schizophrenia

**Figure 1:**
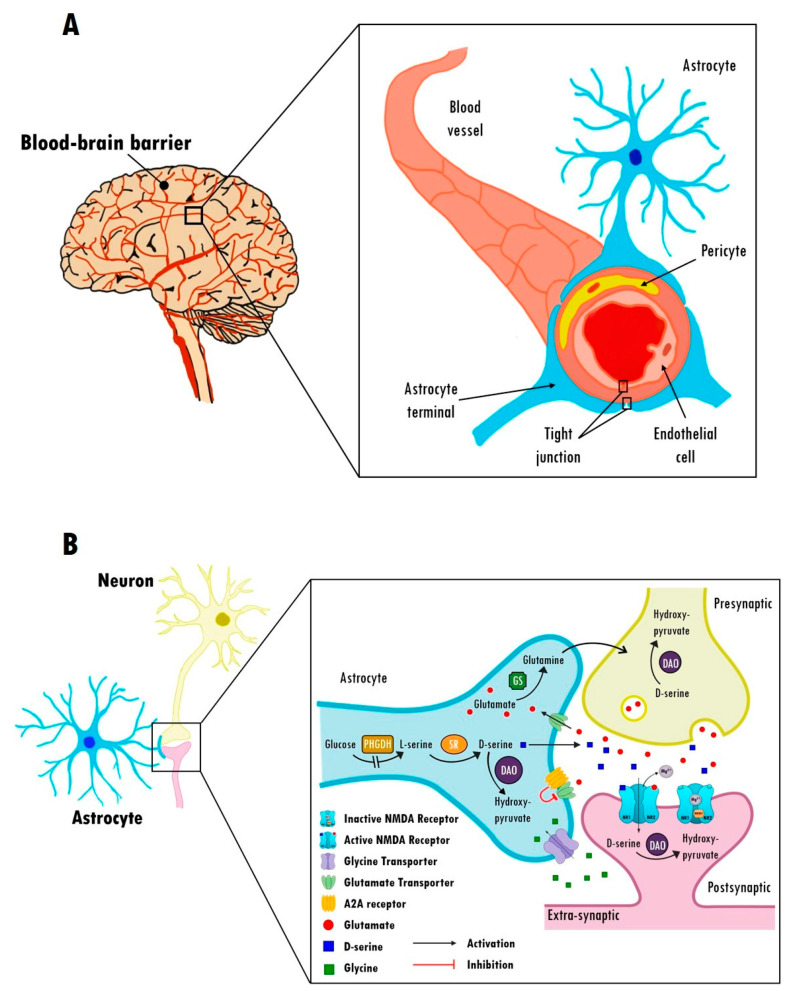
Schematic depiction of the cellular constituents of the blood–brain barrier (BBB) and a model of glutamate trisynapses with presynapses, postsynapses, and astrocytes. (**A**) The BBB is formed by capillary endothelial cells and pericytes and surrounded by astrocytes. The complex tight junctions of endothelial cells and astrocytes contribute to severely restricted penetration and protect against circulating toxins or pathogens in the brain. (**B**) In glutamatergic trisynapses, glutamate is released from glutamatergic presynaptic neurons and binds to the GluN2 subunit of NMDARs postsynaptically. Meanwhile, the GluN1 subunit of the NMDARs is occupied by D-serine or glycine, which is released from astrocytes as coagonists to activate NMDARs. The activation of NMDAR removes Mg^2+^ and opens the ion channel. In astrocytes, glucose is converted to L-serine via the glycolysis pathway with 3-phosphoglycerate dehydrogenase (PHGDH), and then serine racemase (SR) converts L-serine to D-serine, which is released into the synaptic cleft. D-Serine is also metabolized by D-amino acid oxidase (DAO) to hydroxypyruvate in astrocytes. In addition, astrocytes also participate in the glutamate–glutamine cycle. Glutamate is taken up by astrocytes and readily converted into glutamine by glutamine synthetase (GS). In return, glutamine is released into the extracellular space and taken into presynaptic terminals. Astrocytic adenosine A2A receptors regulate excitatory amino acid transporter expressions and glutamate uptake.

As illustrated in Figure 1A, astrocytes play a crucial role in forming the BBB along with pericytes and capillary arterial endothelial cells in the human brain. The BBB separates the human brain from circulating pathogens or metabolized drugs, transports nutrients and secretes growth factors into neuronal cells [33,34], and maintains homeostasis of ions, neurotransmitters, or hormones [35]. The integrity of the BBB relies on tight junctions of endothelial cells and astrocytes connected to neighboring endothelial cells [36]. In particular, astrocytes surrounding the BBB serve as a second barrier to limit the passage into the brain of molecules present in the blood [36]. Accumulated studies have indicated that BBB leakage is detected in patients with mild cognitive impairment or dementia [37,38], suggesting that there is a general link between BBB impairment and the development of cognitive impairment. The increase in BBB permeability has also been considered a biomarker of cognitive dysfunction in Alzheimer’s disease [39,40]. Animal studies further showed the strong relationship between BBB impairment and cognitive deficits, including learning [41] and memory [42].

Currently, the gold standard technique for quantifying BBB integrity in humans is measurement of the CSF/serum albumin ratio (QAlb). Albumin is the primary CSF protein, and its concentration is normally 200 times lower than that in blood. An increased QAlb suggests that increased quantities of albumin can pass from blood to CSF because of an impaired barrier. Similar to those findings of cognitive dysfunction in Alzheimer’s disease, many studies have shown increased QAlb in patients with schizophrenia [43,44,45,46,47,48]. In addition to the QAlb measurement, impairment of the BBB was also reported in the blood of patients. In contrast to nearly undetectable S100β (an astrocytic calcium-binding peptide and an index for BBB dysfunction) in the serum of healthy individuals, increased serum S100β protein levels were found in medication-free schizophrenic patients [49]. Studies of the postmortem human brain also revealed a reduction in claudin-5, a tight junction-associated protein, in the hippocampus of patients with schizophrenia [26,50]. Thus, the impairment of the BBB caused by the dysfunction of astrocytes would be a possible mechanism underlying the cognitive deficits of schizophrenia, which is worthy of further investigation.

### 2.4. Astrocytic Regulation of Glutamate Transmission in Schizophrenia

In addition to various biological functions, astrocytes also have a fundamental role in glutamate metabolism and neurotransmission. Glutamate is the main excitatory neurotransmitter in the nervous system. Since the discovery of the main glutamate receptor, N-methyl-D-aspartate receptor (NMDAR), in the early 1950s, NMDAR has been implicated in many cognitive functions, including learning and memory [51]. All NMDARs are heteromeric complexes composed of a combination of distinct subunits, including GluN1, CluN2, and GluN3 subunits. As illustrated in Figure 1B, the GluN1 subunit is a strychnine-insensitive site for coagonists D-serine and glycine, and the GluN2 subunit is the agonist binding site for glutamate [52]. NMDARs have a glycine modulatory site (GMS) on GluN1 that must be occupied by glycine/D-serine for glutamate to open the channel. The availability of D-serine depends upon the activities of serine racemase (SR), which converts L-serine to D-serine, and the degrading enzyme D-amino acid oxidase (DAO/DAAO) in astrocytes [53], whereas the availability of glycine in the brain is determined by the activity of the glycine transporter (GlyT1) [54,55,56].

Glutamatergic transmission in the vertebrate brain requires the involvement of astrocytes in a continuous molecular dialogue. Astrocytic glutamate receptors and transporters are key molecules that sense synaptic activity and modify their physiology in the short and long terms [57,58]. After glutamate transmission to astrocytes, glutamine synthetase converts glutamate into glutamine. For example, selective removal of oligodendrocyte glutamine synthetase in mice led to a reduction in brain glutamate/glutamine levels and impairment of glutamatergic synaptic transmission [59]. Astrocytes also participate in indirect modulation of NMDARs. Neuronal and glial D-serine production requires astrocytic L-serine generated by a 3-phosphoglycerate dehydrogenase (PHGDH)-dependent pathway [60,61]. Astrocyte-derived L-serine shuttles to neurons and fuels the synthesis of D-serine by SR [62]. Dysfunction of PHGDH reduces the synaptic concentration of D-serine [63]. Furthermore, the termination of glycine-mediated synaptic activity through removal of the neurotransmitter from the synaptic cleft also requires specific GlyT1 present in astrocytes [64].

In addition, astrocytic purinergic system also plays an important role in the modulation of glutamatergic signaling. Adenosine acts as a neuromodulator and exerts different functions depending on the type of adenosine receptors and the consequent cellular signaling involved. The increased adenosine concentration has demonstrated its ability to biphasically modulate the evoked release of glutamate from striatal nerve terminals of rats [65]. Adenosine receptors may regulate excitatory amino acid transporter expressions and glutamate uptake in astrocytes. Intriguingly, selective deletion of adenosine A2A receptors from astrocytes in mice disrupts glutamate homeostasis, leading to psychomotor and cognitive impairments, suggesting that adenosine A2A receptors modulate glutamate signaling and thereby influence some psychomotor and cognitive processes associated with schizophrenia [66]. Additionally, adenosine, by acting on adenosine A1 and A2A receptors, has been reported to antagonistically modulate dopaminergic neurotransmission and is therefore involved in psychostimulant-mediated effects, including locomotor activity, discrimination, seeking, reward, and psychostimulant addiction [67]. In contrast, adenosine A2B receptors are present in astrocytes, neurons, and microglia, but their role in the CNS is less well characterized. These studies support that the dysfunction of the astrocytic purinergic system may trigger an astrocyte-to-neuron wave of communication resulting in disrupted glutamate homeostasis and dopamine neurotransmission, thought to contribute to some endophenotypes relevant to schizophrenia.

Compared to the long-lasting dopamine hypothesis of schizophrenia, the glutamate hypothesis of schizophrenia has become more promising in recent decades. The glutamate hypothesis of schizophrenia was originally proposed based on the observation that NMDAR antagonists (e.g., phencyclidine and ketamine) induced in healthy individuals positive and negative symptoms that resembled schizophrenia [68,69]. Since then, supporting evidence from abnormal expression of NMDAR subunits and NMDAR dysfunction has been implicated in the pathophysiology of schizophrenia [70]. For example, MK-801, a noncompetitive NMDAR antagonist, triggered strong psychomimetic effects with hallucinations and other psychomotor signs that resemble schizophrenia [71]. Given that released glutamate is taken up by surrounding astrocytes, impaired astrocytes can lead to dysfunction of the glutamatergic transmission system and hypofunction of NMDARs in schizophrenia. Postmortem examinations revealed that the cortical expression of astrocytes, glutamate type II, aquaporin-4, S100B, glutaminase, GLT-1, thrombospondin, GFAP, and glutamine synthetase was significantly reduced in the brains of patients with schizophrenia [72,73], supporting the possibility of astrocyte-involved glutamatergic dyshomeostasis in these patients. In addition, evidence suggests that abnormalities in the localization and function of astrocytic glutamate transporters may underlie a disease mechanism with pathological glutamate spillover as well as alterations in the kinetics of perisynaptic glutamate buffering and uptake, which might contribute to the pathophysiology of schizophrenia. An increase in transporter expression in neurons reportedly compensates for the loss of transporter expression in astrocytes [74], which further suggests a profound abnormality in astrocyte functions and their interactions with neurons in the pathophysiology of schizophrenia. Accordingly, the abovementioned studies highlighted two fundamental roles of astrocytes and their involvement in cognitive dysfunctions and schizophrenia. However, the precise underlying mechanism and causal relationship between astrocytes and cognitive dysfunction in schizophrenia still need further elucidation.

## 3. Taking Advantage of Mouse Models and Experimental Tools to Study Astrocytes and Cognitive Deficits in Schizophrenia

**Figure 2:**
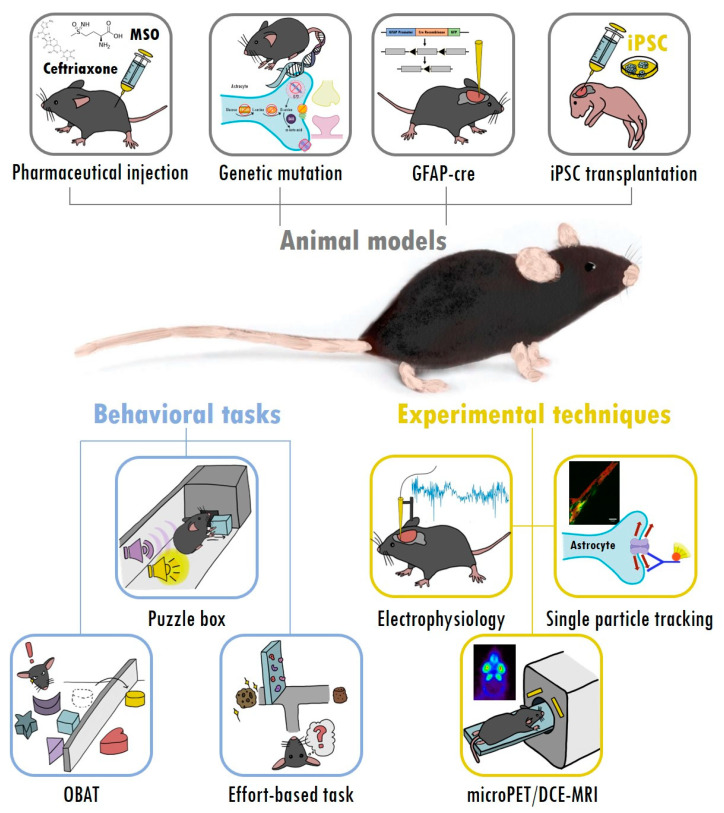
A selection of experimental tools for investigating the role of astrocytes in cognitive deficits in schizophrenia. (The upper panels) Astrocyte-related animal models of schizophrenia, including (from left to right) drug-induced models (such as methionine sulfoximine (MSO) and ceftriaxone injections), genetic mutant mouse models (such as *G72*, *SR*, *PHGDH*, *GlyT1* mutant mice, and *Gfa2-A2AR* knockout mice), *GFAP*-cre mice, and human iPSC transplantation mice. (The lower left panels) A selection of three behavioral tasks for assessing schizophrenia-like cognitive deficits and negative symptoms in animal models. The puzzle box and object-based attention test (OBAT) can be used to assess cognitive function. Effort-based tasks can be used to measure negative symptoms. (The lower right panels) A selection of four experimental techniques to examine the function of astrocytes. Electrophysiological recording is useful for understanding astrocyte-mediated neuronal transmission and oscillation. Single particle tracking can be used to record the trajectory of receptors or proteins on astrocytic membranes. The specific PET radiotracers ^18^F-FDG and ^11^C-DED quantify astrocyte-related physiological, biochemical, and functional processes. Dynamic contrast-enhanced MRI (DCE-MRI) can be used to assess BBB integrity.

Complementary to human studies, applying genetic mutations or pharmacological treatments in animal models provides an efficient opportunity to understand the underlying mechanisms and causal relationship of the pathophysiology of schizophrenia [75,76]. Appropriate animal models with suitable experimental tools provide opportunities to investigate behavioral, functional, structural, and molecular abnormalities at different stages of diseases [75,77]. Currently, along with drug-induced models of schizophrenia, the construction of mice with targeted mutations via gene knockout or transgenic techniques has demonstrated the ability to uniquely identify the functional significance of the targeted gene and its encoded protein with etiological validity [78,79]. As illustrated in Figure 2, a selection of animal models for schizophrenia and experimental techniques was highlighted and introduced to investigate the involvement and roles of astrocytes in the cognitive deficits of schizophrenia.

### 3.1. Using Animal Models to Investigate Astrocytic Regulation of Glutamate Transmission in Schizophrenia

Astrocytes regulate multiple processes in the brain and serve as an integration hub for homeostasis to maintain neuronal functions and modulate metabolic exchange through the BBB [80]. It is therefore not surprising that astrocyte dysfunctions are involved in behavioral abnormalities resembling schizophrenia and in the pathogenesis of some clinical symptoms of schizophrenia. Converging evidence indicates that astrocytes are essential and dynamic partners in both glutamatergic and GABAergic neurotransmissions in the brain [81]. The maintenance of these neurotransmitter pools is strictly dependent on the de novo synthesis of glutamine in astrocytes. As illustrated in the upper panels of Figure 2, the drug-induced and genetically engineered mouse models of astrocyte dysfunction provide a straightforward approach for advancing our understanding of the roles of astrocytes in schizophrenia, and they allow us to directly alter glutamate signaling through manipulation of glutamate, D-serine, and glycine [80]. Indeed, one of the major functions of astrocytes is glutamate uptake, which plays a vital role in the synaptic transmission of glutamate. Glutamate is taken up via glutamate transporter-1 (GLT-1) and converted to glutamine by glutamine synthetase (GS) in astrocytes. Abnormalities of GLT-1 and GS were found in the prefrontal cortex of schizophrenic patients [82,83,84]. Rats treated with ceftriaxone, a GLT-1 activator, displayed increased expression and activity of GLT-1 in astrocytes and impaired sensorimotor gating function, learning, and memory [85,86]. Mice treated with methionine sulfoximine injection (an inhibitor of GS) displayed hypoactive GS activity, resulting in low active glutamatergic signaling in the CA3 region of the hippocampus and impaired spatial memory function [87]. Moreover, astrocytic adenosine receptors A2A play an important role in regulating glutamate uptake and preventing glutamate accumulation [66,88]. A selective deletion of A2A receptors in astrocytes in mice (i.e., *Gfa2-A2AR* knockout mice) resulted in disrupted glutamate homeostasis, which underlies several endophenotypes relevant to schizophrenia [66]. These *Gfa2-A2AR* knockout mice exhibited aberrant GLT-1 activity, increased presynaptic glutamate release, GluN2B upregulation, increased internalization of AMPA receptors, as well as enhanced MK-801 psychomotor responses and decreased working memory. These studies highlight the importance of glutamate and support the involvement of astrocytes in the pathogenesis of schizophrenia. The precise role of glutamate transmission in astrocytes and its involvement in cognitive deficits in schizophrenia are worth further investigation.

In addition to glutamate, D-serine and glycine act as coagonists of the NMDAR glycine modulatory site and contribute to the pathophysiology of schizophrenia [89]. GlyT1 is the major glycine transporter subunit at glutamatergic synapses and is expressed in presynaptic and postsynaptic terminals and astrocytes, contributing to decreased extracellular glycine concentrations at the synaptic cleft [90,91]. *GlyT1*-deficient mouse models have been used to evaluate the preclinical *in vivo* efficacy of drug candidates for enhancing NMDAR function in the treatment of schizophrenia-like symptoms, especially cognitive-related deficits [90,92,93,94]. In addition, as mentioned previously, glucose is converted to L-serine via the glycolysis pathway with PHGDH [60,95], and then SR converts L-serine to D-serine [96]; meanwhile, D-serine is metabolized by D-amino acid oxidase (DAO) [97] in astrocytes. A previous study indicated that a PHGDH antagonist inhibits NMDAR synaptic activation in the hippocampal CA1 region of the mouse brain [62], and PHGDH knockout in mice leads to serine and glycine deficiency and embryonic lethality [98]. Moreover, *PHGDH* conditional knockout mice were not embryonic lethal; however, microcephaly, decreased L-serine, D-serine and glycine in astrocytes, and impaired LTP and spatial memory were observed [63,99]. Alternatively, although recent studies report that SR and DAO are not solely expressed in astrocytes [100,101,102], astrocytes play a vital role in the secretion of D-serine and the regulation of NMDAR function [96,103]. Several animal studies with constitutive deletion of *SR* in *SR^–/–^* mutant mice indicated that these mice had long-term D-serine deficiency, neurobiological abnormalities, spatial and fear memory deficits, sociality problems, and hyperlocomotion activity [104,105,106,107,108]. Intriguingly, a recent study further indicated that *SR^–/–^* mutant mice exhibited a reduction in the freezing response in the trace fear conditioning task and hyperlocomotion in the open-field task, which were normalized by a single administration of sarcosine (an astrocytic glycine transporter-1 inhibitor) [108]. However, little is known about NMDAR hypofunction induced by DAO overexpression in animal models.

In contrast, the G72 protein, also known as a DAO activator, has been detected in astrocytes and regulates DAO activity and D-serine levels [109,110]. Specifically, *G72* transgenic mice exhibited deficits in motor coordination, social ability, prepulse inhibition (PPI), learning and memory, and synaptic transmission in the hippocampus, all of which reflect the symptoms of schizophrenia [111,112,113]. Furthermore, *GFAP*-cre mice and human iPSC transplantation mice represent advanced models that focus on the causal relationship between astrocytes and schizophrenia. For example, taking advantage of *GFAP*-cre mice, mice with deletion of both interleukin-6 and interleukin-6 receptors in astrocytes displayed alterations in locomotor activity, anxiety, and exploratory behaviors [114]. Mice transplanted with schizophrenia iPSCs show delayed astrocytic differentiation, abnormal astrocytic morphologies, and reduced PPI as well as schizophrenia-related behavior [32]. Collectively, apart from the neuronal aspect of schizophrenia, these astrocyte-related animal models provide a better understanding of the pathogenesis of schizophrenia from the aspect of astrocytes and provide additional therapeutic targets for the treatment of schizophrenia.

### 3.2. A Selection of Behavioral Tasks for Assessing Cognitive Deficits and Negative Symptoms of Schizophrenia in Animal Models

Schizophrenia is a psychiatric disorder in humans. Generating “ideal” animal models that recapitulate the full phenotypic spectrum of schizophrenia is nearly impossible. Although we are still far from understanding the precise etiologies and pathogenesis of schizophrenia, many appropriate experimental designs and behavioral tasks in animal models can help clarify and answer these questions. Conventional behavioral tasks have been developed or adopted to evaluate schizophrenia-relevant behavioral phenotypes in mouse models [75,76,77,115]. For example, animals administered psychostimulants in the open field test can be tested using psychomotor agitation for positive symptom-related behavior; social preference tasks and sucrose preference tasks are relevant to sociality and anhedonia, respectively, which have been used to investigate negative symptom-related behaviors; and cognitive dysfunction in schizophrenia, such as sensorimotor gating deficits or learning and memory problems, can be examined in the PPI and Morris water maze, respectively. To date, pharmacological treatments for negative and cognitive symptoms of schizophrenia are still unmet medical needs. Consequently, here, we focus on the negative and cognitive symptoms and highlight three specific behavioral tasks for measuring schizophrenia-related cognitive deficits in mice, including problem solving, attention, and motivation, as illustrated in the lower left panels of Figure 2. These specific cognitive functions were identified by the Measurement and Treatment Research to Improve Cognition in Schizophrenia (MATRICS) [116] and Cognitive Neuroscience Treatment Research to Improve Cognition in Schizophrenia (CNTRICS) criteria [117]. They are of particular interest for the measurement of specific core features of schizophrenia. Moreover, deficits in these cognitive functions have also prompted researchers to develop cognitive remediation therapy for the treatment of patients with schizophrenia [118]. Indeed, in a recent systematic review and meta-analysis, cognitive remediation was confirmed as effective on both cognitive and functional outcomes and potentially useful for all patients with schizophrenia, even those most severely affected [119]. Thus, in complementary to human studies, it is worth investigating and developing animal models representing and mimicking cognitive processes in humans.

Cognitive impairments are considered a core feature of schizophrenia [120]. Problem-solving ability (or executive function) is an important cognitive ability, but it is challenging to measure in animals. Inspired by mental rotation and perseverance tasks in humans, a puzzle box originally designed for rats in 1995 [121] and later modified for mice [122,123] is a simple and efficient behavioral task for evaluating general cognition and executive functions in mouse models of schizophrenia [124,125]. Briefly, each experimental mouse was placed in an apparatus divided into two compartments: a brightly-lit, spacious start zone with loud noises and a smaller covered goal zone without a noise. In each trial, each mouse started from the start zone and then went into the goal zone through a narrow underpass blocked by different obstructions in order to escape from the bright lights and loud noises. Following nine trials over three consecutive days with increased difficulties, it was possible to measure problem-solving ability and short-term and long-term memory in mice [124]. Previous studies demonstrated that both MK-801-treated mice and GluN1 subunit knockdown mice exhibited deficits in the puzzle box task, indicating that the puzzle box is an effective screening tool for investigating executive functions in NMDAR hypofunction mouse models of schizophrenia [124,125].

In addition, attention impairment is also commonly observed among schizophrenic patients and individuals with genetic risks for schizophrenia [2,126]. The 5-choice serial reaction time task (5-CSRTT) is widely used to measure attentional functions in rodents, and it has been applied to model attentional dysfunction in schizophrenia [115,127]. However, there are some drawbacks of 5-CSRTT, including long training sessions, vast experimenter effort, chronic stress in experimental animals, etc. [128]. Many researchers need an efficient behavioral task that assesses attentional functions to be developed. Taking advantage of the natural tendency in mice to explore novelty, an object-based attention (OBA) task was established as a simple and practical behavioral task for evaluating attentional function in mice [129,130]. In this task, mice were first allowed to explore five different but similar objects in the exploratory chamber for a 3 min training phase, and then they were immediately transferred to the test chamber where they explored one familiar object and one novel object during a 3 min testing phase. The time spent exploring each object was recorded in each chamber, and a recognition (i.e., novel vs. familiar objects) index was calculated to indicate attentional function. For verification, the OBA task was confirmed in an attention deficit model induced by acute phencyclidine (1 mg/kg, i.p.) treatment in mice [129]. It is implied that not only can the OBA task assist the behavioral screening process of pharmacological studies on attention-improving drugs but also can evaluate attentional functions in mouse models of schizophrenia. Moreover, unlike the currently available tasks for assessing attention with food/liquid reinforcers, the response bias and uncertainty in the result can be reduced in the OBA task.

Additionally, motivation is a complex and multifaceted process that induces behavioral activation to exert the effort necessary for survival [131]. Previous studies have revealed that motivation-induced behavioral activation is relevant for investigating psychopathology [131]. Indeed, avolition, lack of motivation, is characterized as a negative symptom of schizophrenia [132]. Thus, the behavioral tasks of motivational dysfunctions could foster pathologies and lead to drug treatment for negative symptoms of schizophrenia. Over the past few decades, an emerging area of research has focused on effort-based decision making. Currently, two effort-based decision-making tasks are available, including the concurrent lever-pressing/chow-feeding choice task [133,134] and the T-maze barrier choice task [135,136]. In the concurrent lever-pressing/chow-feeding choice task, each animal was allowed to choose either a lever with a progressive ratio for high rewards or another lever for free approach to low rewards. Similarly, in the T-maze barrier choice task, each animal was allowed to choose either a runway with a vertical barrier for a large amount of reinforcement or another runway with no barrier for a lower amount of reinforcement. As the task difficulty increased, the motivation to choose the high effort option declined in these mice/rats. Both the mesostriatal DA pathway and the glutamate and GABA systems are involved in the process of motivation [131,137,138]. Thus, effort-based decision-making tasks provide a suitable behavioral measurement for investigating the neural mechanisms underlying motivation and the involvement of astrocyte dysfunction in cognitive deficits.

### 3.3. Experimental Tools to Evaluate the Function of Astrocytes

In addition to the abovementioned specific behavioral tasks, multiple analytical tools and neurobiological techniques provide further insight into astrocyte dysfunction and its involvement in the pathophysiology of schizophrenia. As illustrated in the lower right panels of Figure 2, four powerful techniques for investigating biomolecular functions and structures are highlighted here as examples. First, analysis of electrophysiological endophenotypes of NMDAR-mediated transmission is a widely used method for understanding pathological neuronal networks and tripartite synaptic signal integrations [139,140]. For example, sarcosine, an astrocytic glycine transporter-1 inhibitor, enhanced NMDAR-mediated field excitatory postsynaptic potentials and bound to the GMS of NMDARs in the CA1 region of mouse hippocampal slices [108]. Both in vitro electrophysiological studies [29,141,142] and in vivo experiments [29,143,144] with cell type-specific recordings were proven to be useful in revealing that astrocytes mediate neuronal transmission and oscillation. Second, molecular imaging with positron emission tomography (PET) provides the quantification of diverse physiological, biochemical, and functional processes in schizophrenic patients and animal models [108,145,146,147,148]. Intriguingly, the most commonly used PET radiotracer, ^18^F-FDG, is driven by astrocytic glutamate transport [149]. For example, ^18^F-FDG PET/CT brain images revealed that the injection of MK-801 increased overall brain activity, and treatment with sarcosine significantly normalized MK-801-induced abnormalities in the mouse brain [108]. In addition, the best-known PET radiotracer for astrocytes is ^11^C-deuterium-L-deprenyl (^11^C-DED), and it has been employed as a biomarker of astrocytosis in pathologies such as Alzheimer’s disease [150,151]. ^11^C-DED is an irreversible monoamine oxidase B (MAO-B) inhibitor, and the potential application of ^11^C-DED MRI has been demonstrated in detecting astrocyte function in patients with schizophrenia [152,153]. Third, to noninvasively assess the integrity of the BBB, an innovative neuroimaging technique called dynamic contrast-enhanced MRI (DCE-MRI) has also been developed. BBB breakdown could be seen on DCE-MRI by the extravasation of contrast medium into the brain parenchyma. Moreover, DCE-MRI can also be used to detect BBB integrity in animal disease models, such as stroke [154], traumatic brain injury [155], and Alzheimer’s disease [156]. The considerable technical progress of DCE-MRI in combination with PET scans provides not only BBB permeability measurements but also information on CNS transport and lymphatic drainage in the CNS [157]. This system demonstrated an alternative method for exploring the pharmacokinetic aspects of chemical entities with CSF transport through the BBB. However, DCE-MRI has not been applied to schizophrenia in either humans or animals. While techniques for visualizing the BBB are emerging, there is a great chance to deduce the specific role of astrocytes and BBB integrity in various neuropsychiatric disorders. Last, single particle tracking is a revolutionized tool for interrogating dynamics in live cells because of its ability to reveal dynamics in the structure–function relationships underlying the heterogeneous nature of such systems [158,159]. For example, taking advantage of single particle tracking on the GluN1 subunit of NMDARs, a recent study indicated the surface dynamics and movement of NMDARs before and after sarcosine treatment in cultured hippocampal neurons from 18-day-old rat embryos [108]. Single particle tracking techniques with specific quantum dots can be used to record the trajectory from the motion of receptors or proteins on astrocyte membranes [160,161,162] and the behaviors of membrane molecules, which reflect the condition of astrocytes in pathological disease states [163]. Collectively, the abovementioned techniques are worth applying to explore the pathogenesis of astrocyte dysfunction in animal models of schizophrenia and to further develop effective therapeutics for the treatment of schizophrenia.

## 4. Summary and Conclusions

Unfortunately, the etiology and pathophysiology of schizophrenia remain unclear. Pharmacological treatment for negative symptoms and cognitive deficits of schizophrenia has become an unmet medical need. Apart from investigating the neural mechanisms underlying schizophrenia, emerging evidence has begun to reveal the importance and involvement of astrocytes in schizophrenia, especially with regard to cognitive functions. A selection of animal models and experimental tools have been introduced and highlighted for investigating the role of astrocytes in the pathogenesis of schizophrenia and cognitive dysfunctions. A selection of three behavioral tasks is recommended for investigating schizophrenia-related cognitive deficits (i.e., puzzle box for problem solving and OBA test for attentional function) and negative symptoms (i.e., effort-based decision-making tasks for motivation) in animal models. Here, we described the current knowledge pertaining to astrocytes’ role in the pathogenesis of schizophrenia and introduced useful experimental models and tools to further elucidate the role of astrocytes in the cognitive deficits of schizophrenia. Collectively, emerging evidence suggests that astrocytes are not just bystanders but are involved in the pathophysiology of schizophrenia. Given that astrocytes participate in a variety of essential physiological processes in the CNS, it would also be beneficial to use these astrocyte-related animal models and experimental tools to investigate their involvement in other neuropsychiatric disorders, as reported previously for Rett syndrome [164], Fragile X syndrome [165], major depressive disorder [166], bipolar disorder [167], Alzheimer’s disease [168], Prader–Willi syndrome [169], Parkinson’s disease [170], etc. The precise role and underlying mechanism of astrocytes are worth further investigation.

## Data Availability

Not applicable.
